# Pre-emptive Intervention for Autism Spectrum Disorder: Theoretical Foundations and Clinical Translation

**DOI:** 10.3389/fnint.2019.00066

**Published:** 2019-11-19

**Authors:** Pamela S. Douglas

**Affiliations:** ^1^Transforming Maternity Care Collaborative, Griffith University, Brisbane, QLD, Australia; ^2^Discipline of General Practice, The University of Queensland, Brisbane, QLD, Australia

**Keywords:** autism (ASD), pre-emptive, intervention, environmental factors, complex adaptive systems

## Abstract

Autism spectrum disorders (ASD) are an emergent public health problem, placing significant burden upon the individual, family and health system. ASD are polygenetic spectrum disorders of neural connectome development, in which one or more feedback loops amplify small genetic, structural, or functional variations in the very early development of motor and sensory-motor pathways. These perturbations trigger a ‘butterfly effect’ of unpredictable cascades of structural and functional imbalances in the global neuronal workspace, resulting in atypical behaviors, social communication, and cognition long-term. The first 100 days post-term are critically neuroplastic and comprise an injury-sensitive developmental window, characterized by a neural biomarker, the persistence of the cortical subplate, and a behavioral biomarker, the crying diathesis. By the time potential diagnostic signs are identified, from 6 months of age, ASD neuropathy is already entrenched. The International Society for Autism Research Special Interest Group has called for pre-emptive intervention, based upon rigorous theoretical frames, and real world translation and evaluation. This paper responds to that call. It synthesizes heterogenous evidence concerning ASD etiologies from both psychosocial and biological research literatures with complexity science and evolutionary biology, to propose a theoretical framework for pre-emptive intervention. This paper hypothesizes that environmental factors resulting from a mismatch between environment of evolutionary adaptedness and culture initiate or perpetuate early motor and sensory-motor lesions, triggering a butterfly effect of multi-directional cascades of atypical developmental in the complex adaptive system of the parent and ASD-susceptible infant. Chronic sympathetic nervous system/hypothalamic-pituitary-adrenal axis hyperarousal and disrupted parent-infant biobehavioral synchrony are the key biologic and behavioral mechanisms perpetuating these atypical developmental cascades. A clinical translation of this evidence is proposed, for application antenatally and in the first 6 months of life, as pre-emptive intervention for ASD.

## Background

### What Is Autism?

Autism spectrum disorders (ASD) are a group of complex and heterogenous neurodevelopmental conditions, with life-long presentations in behavior, communication, cognition, and mental health. They are defined behaviorally, by early onset of persistent difficulties in social communication and interaction, sensory atypicalities, and repetitive, restricted interests and activities causing impairment in daily life. Due to the multiple and interdependently variable traits that pertain to autism, Autism-Related Disorders has been recently proposed as a more accurate clinical descriptor (Greenspan, 2018). ASD presents differently in males and females, in a ratio of 4:1. Traits of autism are continuously distributed in the general population and overlap with clinical phenotypes.

Autism spectrum disorders is diagnosed in one in 68, or 0.7%, of Australians. Prevalence has increased across all age groups between 2009 and 2015 in Australia, most markedly in the 5–14 years age group, consistent with international trends. This rapid increase is attributed to increased awareness and diagnosis. Although signs may emerge much earlier, most children are not diagnosed until at least 2 or 3 years of age, and the mean age of diagnosis in Australia is 4.5 years (Synergies Economic Consulting, 2011).

The total direct and indirect cost of ASD annually was estimated at $5.8 billion in Australia in 2010, with the high cost of treating ASD and its associated co-morbidities subject to intense media scrutiny during the initial roll out of the National Disability Insurance Scheme (Synergies Economic Consulting, 2011). The cost to the health system over the life-span for each individual diagnosed with ASD has been estimated at $2.4 million in the United States, or £1.5 million in the United Kingdom, higher than for asthma or diabetes (Buescher et al., 2014).

#### Etiology

Autism spectrum disorder are polygenic syndromes, associated with 102 genes in a 2018 genetic sequencing study, and also with *de novo* mutations (De Rubeis and Buxbaum, 2015; Satterstrom et al., 2018). However, any individual’s complex genetic susceptibility is impacted by a myriad of environmental factors in intra-uterine and early life, which alter epigenomic regulation and phenotype expression (Tordjman et al., 2014; Keating, 2016; Mandy and Lai, 2016; Matas et al., 2016; Ismail et al., 2017; Barker et al., 2018).

Autism spectrum disorder is best conceptualized, applying a dynamic systems or complexity science framework, as a spectrum disorder of connectome development, that is, of the neural wiring of the brain, in which one or more feedback loops amplify small variations in very development. Initial lesions trigger a ‘butterfly effect’ of unpredictable cascades of structural and functional imbalances in the global neuronal workspace, which dynamically interact with and impact upon the infant’s social and non-social environmental experiences, amplifying feedback loops and affecting behaviors, cognition, and social communication long-term (Fields and Glazebrook, 2017).

The initial lesions which cause feedback loop disruptions may be structural, either genomic or due to injury; or functional, for example, from changes in the monoaminergic system. Variable phenotypic expressions emerge out of the compensations of the child’s neural networks in response to very early lesions, as myriad feedback loops in the complex adaptive system of the global neuronal workspace compensate for deficiencies and maintain the best possible functional stability. This may occur at the expense of, or with unusual development of and compensation by, higher order cognitive functions like memory, attention and executive functions. The heterogeneity of ASD reflects the multiple different disturbances that can occur along any one of multiple pathways. Dysregulation in any one neural or physiological pathway causes a cascade of events culminating in a cluster of symptoms (Fields and Glazebrook, 2017).

At the cellular level, feedback loop imbalances sculpt neuron morphology and synaptogenesis and alter synaptic transmissions by excitatory or inhibitory neurons. By the time a child is diagnosed with ASD, neuroanatomic patterns of excessive short-range connections and weakened long-range connections have emerged in vulnerable parts of the brain, including in parts of the prefrontal cortex associated with attention, social interaction, emotions, and executive control, and in a decreased density of axons below limbic cortices such as the anterior cingulate cortex (Zikopoulos and Barbas, 2010, 2013; Garcia-Cabezas et al., 2018; Zikopoulos et al., 2018; Trutzer et al., 2019).

Comorbidity of ASD with other medical disorders, including neurodevelopmental, psychiatric, and physical disorders, is common in both children and adults, further supporting the hypothesis that impaired neural and physiological developmental cascades occur in response to very early structural or functional neural lesions. Resultant neural and physiological morbidities then interact with co-occurring behavioral morbidities (Tye et al., 2019).

#### High-Risk Siblings

An infant who has an older sibling diagnosed with ASD is referred to in this paper as a high-risk sibling. An estimated 1 in 5 high-risk siblings will develop ASD (Ozonoff et al., 2011). High-risk siblings who do not develop ASD are nevertheless at risk of suboptimal developmental outcomes (Landa et al., 2012). High-risk siblings who develop ASD comprise only a small proportion of children diagnosed with ASD, and it is unclear to what extent findings from high-risk sibling studies can be generalized to ASD children from simplex families. When 244 two-year-olds diagnosed with ASD were compared, those with an older ASD sibling showed improved cognitive abilities relative to those from simplex families, perhaps because multiplex parents modify their parenting in response to their previous ASD experience (Dissanayake et al., 2019).

#### Autism Prodrome

Research in recent years has focused on identification of an autism prodrome, with the aim of developing effective screening and early intervention. Signs that may be diagnostic of ASD, including repetitive behaviors, delayed motor and language skills, reduced gesturing, and impaired eye gaze patterns, emerge from 12 months of age (Bradshaw et al., 2015; Pierce et al., 2016; Petinou and Minaidou, 2017; Shen and Piven, 2017; Whitehouse, 2017; French and Kennedy, 2018; Green and Garg, 2018; Vivanti et al., 2018). From 6 months of age, high-risk siblings are more likely to show reduced attentiveness to parent, reduced affective signaling, poorer co-ordination of communication, and less use of gestures (Wan et al., 2018). These and other soft signs of neurodevelopmental vulnerability have been identified from 6 months of age in children who are later diagnosed with ASD, and may be evident even earlier (Bolton et al., 2012).

However, it’s not possible to distinguish infants with behavioral signs of neurodevelopmental vulnerability who will be later diagnosed with ASD, from those with behavioral signs of neurodevelopmental vulnerability who will develop other neurodevelopmental disorders (Levit-Binnun and Golland, 2012). Because of the extreme heterogeneity of ASD, the non-specific nature of early signs of neurodevelopmental vulnerability, and the significant risks associated with early diagnosis, the search for clinically useful biomarkers of ASD in the first 6 months of life may not prove realistic (Goldani et al., 2014).

### Pre-emptive and Early Intervention

This paper defines very early life as the first 100 days post-term, and early life as the first 12 months post-term, distinguishing these periods from intra-uterine life. Early intervention is defined here as an intervention for infants showing signs of neurodevelopmental vulnerability or ASD that is implemented between 6 to 18 months of age, and pre-emptive intervention as an intervention for infants less than 6 months of age, whether or not they are showing soft signs of neurodevelopmental vulnerability.

#### The International Society for Autism Research Calls for Rigorous Theoretical Framing and Real World Methodologies

In a 2019 discussion of gaps in ASD research, Bailey laments that:

… *Genetic studies continue to search for risk variants of small effect in complex groups, biological research proceeds in its attempt to find unitary underlying commonalities in members of the ASD category, and early detection research continues to search in vain for markers with clinically useful specificities and sensitivities.*

In this context, Dawson calls for “new and paradigm-shifting interventions and research methodologies which embrace heterogeneity,” and Charman highlights the need to “[transfer] new knowledge about risk factors, biomarkers, and behavioral signs into practical applications” for health professionals (Amaral et al., 2019).

In an era of big data, theoretical framing is key to ongoing knowledge development and cost-effective, sustainable innovation (Campbell et al., 2007; Fox et al., 2014; Ioannadis, 2014). Between 2015 and 2017, the International Society for Autism Research sponsored a Special Interest Group on *Implementing and Evaluating Community-based Early Intervention*. The Special Interest Group calls for:

• Carefully developed theoretical frames to guide early intervention development, evaluation, and implementation;
• An understanding of factors that make interventions more likely to be adopted and implemented in the real world;
• Rapid progression from treatment theory to community-based effectiveness trials, with community-partnered participatory research;
• An iterative approach consistent with the principles of real world research, with multidirectional knowledge exchange occurring between different phases of the research (Vivanti et al., 2018).

Green and Garg observe that large amounts of public and private money are currently being invested internationally in interventions for ASD which lack rigorous theoretical frames or an evidence base, and call for integration of psychosocial and biological intervention models, in order to eludicate potential synergies between promising psychological and biological intervention mechanisms in ASD (Green and Garg, 2018). Green asks that:

… *prodromal intervention should not be tied to legacy theories of the past [but] be responsive to emerging results from basic science to get a more profound understanding of early developmental process and how best to intervene (Green, 2019).*

Here, I aim to respond to these calls, as a primary care medical practitioner who specializes both clinically and as a researcher in the care of parents with infants. This paper integrates existing evidence across multiple disciplines and etiological models, in order to explicate the theoretical frames which underlie a proposed interdisciplinary pre-emptive intervention for ASD, applied from the antenatal period and throughout the first 6 months of life.

The International Society for Autism Research Special Interest Group also calls for isolation and evaluation of ‘active ingredients’ of ASD interventions (Vivanti et al., 2018). But from a complexity science perspective, epigenomic regulation of heterogenous ASD genetic susceptibility is impacted by a myriad of interacting and co-evolving environmental variables (Tordjman et al., 2014; Keating, 2016; Matas et al., 2016; Mandy and Lai, 2016; Ismail et al., 2017; Barker et al., 2018). A reductive approach which aims to identify a small number of active ingredients is, applying complexity science, predicted to have limited real world application. This is because any ingredient or mix of specific selected ingredients (that is, simplified, linear interventions) risk unintended outcomes when applied to complex adaptive systems (Campbell et al., 2007; Braithwaite et al., 2018). This may be one reason why guidelines and interventions designed in university or tertiary settings often lack relevance in community settings (Steel et al., 2014).

A complexity or whole systems approach demands development of a comprehensive and integrated pre-emptive intervention, translated from a rigorous theoretical frame, to address the many environmental factors which co-evolve together and interact with the epigenome, to protect against or mitigate expression of ASD genetic susceptibility. That is, holistic pre-emptive intervention is applied flexibly in order to stabilize as many of the myriad feedback loops within the complex adaptive system of each unique infant’s global neuronal workspace and within the complex adaptive system of the infant’s unique family as early as possible, in order to have the greatest chance of success (Marin, 2016; Green, 2019).

Leading primary care researchers have developed innovative methodologies suitable for the evaluation of complex interventions, since community-based healthcare is typically confronted by complex problems requiring early, complex, cost effective, preventive interventions (Plsek and Greenhalgh, 2001; Campbell et al., 2007; Greenhalgh et al., 2015; Braithwaite et al., 2018).

#### Why Pre-emptive Intervention?

To date, early intervention studies for ASD have been conducted after 6 months of age, either with high-risk siblings or with infants showing signs of neurodevelopmental vulnerability. They derive from applied behavior analysis and social communication models of ASD etiology, applying parent-mediated enrichment of the social interactive environment, and demonstrate preliminary positive outcomes on parent-infant interactions. In particular, evaluation of these early interventions suggest that undemanding synchronous parent behavior, which notices and responds to the child’s focus without suggesting a new action, is associated with stronger joint attention and language skills throughout childhood (Steiner, 2013; Rogers et al., 2014; Baker et al., 2015; Baranek et al., 2015; Bradshaw et al., 2015; Green, 2017; Petinou and Minaidou, 2017; French and Kennedy, 2018; Green and Garg, 2018; Wan et al., 2018; Whitehouse et al., 2019).

However, functional neuroimaging of high-risk siblings who later develop ASD demonstrates altered cortico-cortical connectivity from 6 months of age (Lewis et al., 2017; French and Kennedy, 2018). Regional MRI volumes across the whole brain in 4–6 month old high-risk siblings show larger cerebellar and subcortical volumes than in low risk infants, linked to more repetitive behaviors at 36 months (Pote et al., 2019). These findings suggest that initial changes in the global neuronal workspace occur either prenatally or in very early life. By the time an infant is 6 months old, the effects of very early structural or functional lesions in the global neural workspace are already entrenched, and developmental cascades of multi-directional perturbations in motor, sensory, and social interactions are underway. That is, early interventions evaluated to date have been applied to an already fundamentally altered neural landscape.

Studies of pre-emptive intervention for ASD, as defined in this paper, have not yet appeared in the scientific literature. This paper integrates current understandings of neural network developmental dynamics to support the hypothesis that ASD course alteration is possible, beginning with antenatal anticipatory guidance, followed by intervention in the first months of life.

## The First 100 Days Post-Term: Window of Critically Injury-Sensitive Neuroplasticity

### Neural and Behavioral Biomarkers

Neural network development activity peaks between the last three months *in utero* and the first three months post-birth, spanning from the beginning of cortical subplate diminution until the subplate disappears as permanent circuitries in the primary motor, somatosensory, and visual cortex take over. Until then, sensory-motor information continues to be relayed through subplate neurons, which sculpt permanent cortical templates. Cortical subplate neurons are known to be selectively sensitive to injury, and very early sensory-motor defects due to subplate neuronal injury have been implicated in neurodevelopmental disorders, including ASD (Kanold and Luhmann, 2010; Wess et al., 2017; Hadders-Algra, 2018a).

From 10 weeks post-conception, the brainstem and spinal cord central pattern generator initiates general movements. These disappear by three to 5 months post-birth, as fidgety movements and goal directed, affect-driven movements of the limbs emerge and the cortical subplate disappears. Hadders-Algra notes that the emergence, blooming, and eventual disappearance of general movements coincides temporally with the emergence, dominance, and disappearance of subplate synaptic activity. She hypothesizes that general movement complexity and variability are modulated by the cortical subplate and mediated by subplate motor efferents. In her model, abnormal general movements result from lesions in either the subplate or in subplate motor efferent connections within the periventricular white matter (De Graaf-Peters and Hadders-Algra, 2006; Hadders-Algra, 2007, 2017, 2018a).

Significantly, at the time of birth and in the middle of the period of peak neural network development, noradrenergic alpha 2 receptors and glutamatergic *N*-methyl-D-aspartate receptors are temporarily overexpressed, with high serotonergic innervation and dopaminergic turnover. Hadders-Algra hypothesizes that monoaminergic exuberance from birth until three months of age is associated with increased neural excitability, including of motoneurons. She speculates that monoaminergic exuberance offers evolutionary advantage, and is expressed in the writhing character of general movements. This over-expression resolves as the permanent cortical circuitries take over from the subplate (De Graaf-Peters and Hadders-Algra, 2006; Hadders-Algra, 2007, 2017, 2018a).

Behaviorally, persistence of the cortical subplate and increased monoaminergic excitability correspond post-birth with the infant crying diathesis. The crying diathesis can be viewed, then, as a behavioral biomarker, and the cortical subplate as a neural biomarker, of the critically injury-sensitive neuroplasticity of an infant’s first 100 days of life.

### Stress Response Settings

Neural templates for emotional and stress regulation are laid down in the cortical subplate and cortex, and in subcortical structures such as the basal ganglia and the amygdala, during the developmentally critical first 100 days post-birth. These templates form the basis for secure psychological attachment and mental health life-long. As originally proposed by Greenspan in the Affect Diathesis Model, this paper hypothesizes that chronic sympathetic nervous system and hypothalamic-pituitary-adrenal axis (SNS-HPA) hyperarousal has monoaminergic impacts on the brainstem and cortical subplate during the critical window of monoaminergic excitability and injury-sensitive neuroplasticity, and is a key physiological mechanism disrupting capacity for attention on developmental tasks, mediating atypical developmental cascades (Schore, 2001; Greenspan, 2002; Lydon et al., 2016; Marin, 2016; Mandy and Lai, 2016; Ismail et al., 2017; Patel et al., 2018).

Although genetic susceptibility to long-term HPA axis dysregulation varies, painful perceptual experiences in very early life, including fear or chronic stress (experienced physiologically as chronic SNS-HPA hyperarousal), risk lifetime changes in SNS-HPA axis settings and dopaminergic, serotonergic, or noradrenergic circuitries. The HPA axis does not return to its ‘unstressed’ state, normal patterns of cortisol release fail to emerge, and behaviorally, infants show difficulty moving out of hyper-aroused or hypo-aroused states.

Stress also increases the permeability of the blood brain barrier, allowing penetration of pro-inflammatory cytokines. The immune dysregulation associated with chronic stress and increased allostatic load has widespread effect on physiological systems underlying developmental processes and mental and physical health, including on growth, metabolism, immunity, and cognition (Marin, 2016; Barker et al., 2018; Koss and Gunnar, 2018).

Executive functions and the development of sustained intention, including joint attention, are particularly sensitive to stress. In 12 months old neurotypical infants, those with high autonomic reactivity to stressors show short attention durations, and those with lower autonomic reactivity show longer attention durations (Wass et al., 2018). In older children diagnosed with ASD, stress triggers worsened signs and symptoms, impairing the capacity to achieve developmental tasks, which further upregulates the stress response, perpetuating multi-directional developmental cascades. This paper proposes that the same chronic SNS-HPA hyperarousal known to impact negatively on capacity to perform developmental tasks in the older child similarly impedes an infant’s capacity to perform the vital motor and sensory-motor developmental tasks of very early life.

#### Chronic SNS-HPA Hyperarousal in Very Early Life Is Modifiable by Environmental Factors

The infant crying diathesis is characterized by high levels of cry initiation, which are stable across cultures. In high income countries, the crying diathesis is characterized by durations of cry that average about 2 h a day for the first 6 weeks, decreasing to 72 min a day at 12 weeks, and has resolved, for most, by about 16 weeks post-birth (Wolke et al., 2017). Twenty percent of families report excessive crying, of over 3 h a day, for at least 3 days a week, and many more report cry-fuss problems (Wake et al., 2006). But importantly, infant crying is modifiable according to socioculturally determined environmental factors. Cultures more likely to breastfeed, offer physical contact, and provide cued care have the same high frequency of cry initiation in this period, but at least halved cry durations (St James-Roberts et al., 2006; Wolke et al., 2017).

This paper proposes pre-emptive intervention for ASD which integrates the neurobiological model of cry-fuss problems (Table 1). In the complex adaptive system of parent and infant, problems of crying and fussing, feeds, and sleep often interact and co-evolve, and may be referred to as ‘regulatory problems,’ or ‘dysregulation.’ The neurobiological model of cry-fuss problems conceptualizes problem crying as a behavioral biomarker of chronic SNS-HPA hyperarousal, that is, of stress in very early life (Douglas et al., 2011; Douglas and Hill, 2013a). Crying and fussing, or chronic SNS-HPA hyperarousal, emerge when the parent-infant complex adaptive system is unable to stabilize multiple feedback-loop disruptions arising from interacting and co-evolving environmental factors (Douglas et al., 2011).

**TABLE 1:** Current explanatory models for cry-fuss problems.

**Theoretical model**	**Key management strategies**	**Summary of evidence**
Medical condition		Not supported by evidence (Douglas, 2013;
(1) ‘Reflux’ or GORD	(1) Anti-secretory medications	Bergmann et al., 2014; Gieruszczak-Bialek et al., 2015;
(2) Allergy	(2) Maternal elimination diet	O’Shea et al., 2017; Gordon et al., 2018)
(3) Tongue-tie or upper lip-tie (in absence of classic tongue-tie)	(3) Frenotomy	
(4) Lactose intolerance	(4) Lactose-free formula	
Normal developmental phase (Zeifman and St James-Roberts, 2017)	Support carer coping. Reassure crying will pass Entrain infant biology with first wave behavioral (FWB) strategies.	Ignores evidence that crying durations are modifiable by infant care practices (Wolke et al., 2017). High level evidence shows that FWB strategies do not decrease night waking (Bryanton et al., 2013; Douglas and Hill, 2013b; Kempler et al., 2016; NHMRC, 2017)
‘A mysterious disorder of the microbiota-gut-brain axis’ (Partty and Kalliomaki, 2017; Rhoads et al., 2018; Zeevenhooven et al., 2018)	Probiotics	Probiotics may decrease crying in breastfed infants (placebo response 66%) but studies do not control for the breastfeeding problem of functional lactose overload and do not take into account complex bidirectional nature of gut-brain axis (multiple confounders). Gut dysbiosis is a confounder, not a cause (Fatheree et al., 2017; Sung et al., 2017).
Neurobiological (Douglas and Hill, 2013a)	Neuroprotective Developmental Care, which Integrates lactation and sleep science, neuroscience, brain- gut-microbiota science, evolutionary biology, applied functional contextualism.	Preliminary studies positive (Douglas et al., 2013; Ball et al., 2018) Requires further research.

The neurobiological model of cry-fuss behaviors identifies three key environmental causes of chronic SNS-HPA hyperarousal in the first 100 days post-term:

(1) Suboptimal environmental stimulation
(2) First wave behavioral (FWB) approaches to infant sleep
(3) Unidentified and unmanaged feeding problems, either
  (a) Poor satiety
    (i) due to compromised milk transfer in breastfeeding, typically due to suboptimal fit and hold (‘latch and positioning’), and resultant low supply
    (ii) due to feed-spacing in either breast or formula-fed infants
  (b) Functional lactose overload in breastfed infants
  (c) Conditioned SNS-HPA hyperarousal with feeds, most often due to positional instability at the breast, or coercive practices during either breast or bottle feeds (Douglas and Hill, 2013a).

In the neurobiological model of cry-fuss problems, these disruptive environmental factors interact unpredictably in the context of monoaminergic excitability in the first 100 days of life and predispose to:

(1) High levels of SNS-HPA arousal, which temporarily trigger more SNS-HPA arousal, in a positive feedback loop of inconsolable crying (correlating with monoaminergic excitability);
(2) Development of conditioned SNS-HPA hyperarousal in response to particular triggers e.g., conditioned SNS-HPA hyperarousal with breastfeeding or bottle-feeding;
(3) Temporary re-setting of the baby’s ‘stress thermostat’ in response to chronically high levels of SNS-HPA arousal (also known as ‘difficulty shifting arousal states’), which resolves at the end of the crying period;
(4) Permanent re-calibration of the ‘stress thermostat’ in biologically susceptible infants (that is, permanent alteration of neural connectivity), due to:
  (a) Alteration of the infant’s stress response settings, and/or
  (b) Cascade effects of altered parent-infant interactions;
(5) Maternal chronic SNS-HPA hyperarousal in response to infant crying, feed, and sleep problems, which increases the risk of:
  (a) Postnatal anxiety and/or depression
  (b) Avoidance behaviors (Douglas and Hill, 2013a).

#### Chronic SNS-HPA Hyperarousal in Very Early Life Permanently Alters Stress Response Settings in Susceptible Infants

Multiple studies link infant regulatory problems in the domains of feed, cry-fuss behaviors and sleep with impaired developmental and behavioral outcomes in later childhood. This association is strongest if there is more than one regulatory problem, or if the regulatory problem persists beyond 4–5 months of age (DeGangi et al., 2000; Desantis et al., 2004; Von Kries et al., 2006; Schmid and Wolke, 2014; Winsper and Wolke, 2014; Santos et al., 2015; Laetitia et al., 2016; Sidor et al., 2017; Williams et al., 2017; Bilgin et al., 2018; Breeman et al., 2018; Cook et al., 2019). Regulatory problems in infancy have also been linked with avoidant adult personality traits (Bauml et al., 2018). Multiple persistent regulatory problems are associated with attention problems in later childhood, and trajectories of attention problems from childhood to adulthood (Bilgin et al., 2018). A comparative study which claims to show no link between regulatory problems in the first 3 months of life and behavior problems at 2–3 years of age retrospectively excludes infants whose crying was found to persist at 6 months of age (Bell et al., 2018). Yet it is not possible to predict which infants with cry-fuss problems in very early life will have persistent crying at 5 or 6 months of age, and intervention should be offered promptly to all parents reporting problem crying.

More ‘cued care’ at term predicts fewer regulatory problems at three months, but after the first highly neuroplastic 100 days, patterns of chronic SNS-HPA hyperarousal may become entrenched in susceptible infants, since cued care beyond this age does not result in less regulatory problems. Biological vulnerabilities such as preterm birth provide more significant prediction of long-term regulatory problems (Bilgin and Wolke, 2017). But upregulated infant behavior, or crying and fussing, also predisposes to negative affect and mood problems in parents (Vik et al., 2009; Cook et al., 2017), which disrupt the parent’s capacity to engage and lengthen reciprocity chains. This bi-directional disruption to parent-infant biobehavioral synchrony adds to allostatic load in infants.

These findings corroborate the neurobiological model of infant crying, which argues that while most families are resilient, and suffer no long-term effects of excessive infant crying, cry-fuss problems are a behavioral biomarker of infant stress and disrupted parent–infant biobehavioral synchrony due to modifiable environmental factors, and affect the infant’s capacity to attend to developmental tasks. In a small but important subset of genetically susceptible infants or psychosocially vulnerable families, cry-fuss problems are then associated with long-term SNS-HPA axis disruption and developmental impairment (DeGangi et al., 2000; Desantis et al., 2004; Von Kries et al., 2006; Schmid and Wolke, 2014; Winsper and Wolke, 2014; Santos et al., 2015; Laetitia et al., 2016; Sidor et al., 2017; Williams et al., 2017; Bilgin et al., 2018; Breeman et al., 2018; Cook et al., 2019).

### Primacy of Motor Lesions in Development of ASD

Motor, sensory, cognitive, and social communication development are not independent phenomena occurring in sequential order or in discrete neuroanatomic locations, but are complex functions which dynamically co-evolve within the global neuronal workspace of the complex adaptive systems of the developing fetus and infant (Whyatt and Craig, 2013; Estes et al., 2015; Fields and Glazebrook, 2017).

Up to 60% of children with ASD have motor deficits, including impaired postural control, impaired motor planning and sequencing, which affects gesture planning and imitation, and low motor tone (Paquet et al., 2016; Paquet et al., 2019). Prospective or feedforward mechanisms of motor timing, also referred to as sensory-motor intentionality, are typically disrupted in ASD, as is perceptual awareness of others’ intentions conveyed in body movement or eye gaze. Torres et al. argue that motor disruption is a core feature of ASD, requiring assessment methods suitable for inclusion in clinical diagnostic criteria (Torres et al., 2013).

This paper draws on the hypotheses concerning the primacy of either *in utero*, intra-partum, or very early life motor lesions in ASD etiology, as detailed in ground-breaking syntheses by Torres et al. (2013), Trevarthen and Delafield-Butt (2013), Delafield-Butt and Trevarthan (2017), Dadalko and Travers (2018), and Delafield-Butt et al. (2018). These researchers propose that a structural (genetic or injury) or functional (monoaminergic) motor lesion is sustained in the brainstem systems or cortical subplate in the critically neuroplastic window *in utero*, intra-partum, or in very early life, which impairs prospective, affect-driven movement. Cascades of imbalance between local and global connectivity emerge, resulting in atypical neurological, psychological, and behavioral development. Chronic SNS-HPS hyperarousal and disrupted parent-infant biobehavioral synchrony are key physiological and behavioral mechanisms which either predispose to very early motor lesions, or perpetuate the effects of very early motor lesions.

Infants require complex and unpredictable postural variability from birth in order to optimize postural control strategies; they also require rich and active movement experiences in order to learn to perceive visual and tactile stimuli (Hadders-Algra, 2010; Dusing, 2016; Hadders-Algra, 2017; Hadders-Algra, 2018b). Reduced movement complexity and variability, that is, reduced movement repertoire, associated with decreased affect-driven prospective movement from birth, impairs sensory feedback and parent response, limiting capacity to process sensory information (Delafield-Butt et al., 2018). In the phase of secondary variability of general movements, these infants have further difficulty selecting an appropriately adapted strategy from of their repertoire, due to limited variability (Hadders-Algra, 2010; Dadalko and Travers, 2018; Delafield-Butt et al., 2018; Hadders-Algra, 2018b). Shafer et al. (2017) propose that motor stereotypy is a downstream manifestation of low motor complexity. The reduced exploratory motor drive evident in many ASD children is hypothesized to begin with these deficits in primary and secondary variability of movement, which cascade to motor coordination difficulties and impaired complex motor sequencing (Denisova and Zhao, 2017) (Box 1).

**BOX 1. Selected studies corroborating the hypothesis that very early life motor lesions initiate the atypical developmental trajectories of ASD.**

See Torres et al. (2013), Trevarthen and Delafield-Butt (2013), Delafield-Butt and Trevarthan (2017), Dadalko and Travers (2018), Delafield-Butt et al. (2018) for comprehensive reviews.

A retrospective 2008 study of videos of 20 children diagnosed with ASD demonstrated impaired complexity and variability of general movements and absent or abnormal fidgety movements in infancy (Phagava et al., 2008). A 2013 prospective, longitudinal study of 235 low and high-risk infants aged 6–36 months demonstrated developmental differences from 6 months of age, and poorer fine motor skills were evident in the high-risk infants by 14 months of age (Landa et al., 2013; Bradshaw et al., 2018). In a 2014 study of 158 high-risk siblings and low risk infants, repetitive and stereotyped motor behavior was observed as early as 12 months of age in high-risk siblings who are were diagnosed with ASD at 24 months of age (Elison et al., 2014). A 2017 comparative study of 71 low and high-risk infants showed that high-risk siblings who later develop learning delays have less variety of general movements in the first 8 weeks post-birth, and less variety of movements in response to language, suggesting less flexible sensory-motor systems (Denisova and Zhao, 2017). A 2018 study comparing 86 high-risk siblings with 113 low risk infants showed that high-risk siblings who walked independently at 12 months of age had superior social-communication skills relative to high-risk siblings who weren’t walking. A 2019 prospective study of 437 high-risk siblings and 188 low risk infants were assessed at 6 and 36 months. High-risk siblings have poorer fine motor skills and delayed motor development at 6 months, though these findings are not predictive of later diagnosis of ASD (Iverson et al., 2019).

The social communication developmental pathway depends throughout early life on motor competency and capacity for motor synchrony with another, including mutual gaze, joint attention and shared positive affect, attention disengagement, gesture, touch, and language learning (Yu and Smith, 2017). For example, from birth multiple sequential motor and sensory-motor patterns lead to joint attention. By three months of age, social gaze is the primary modality of coordinated interactions, occurring 30–50% of the time in low risk infants. But attention to eyes declines between 2 and 6 months in infants who later develop ASD (Jones and Klin, 2013), and high-risk siblings perform lower on visual attention tasks at 2 and 3 months of age compared to low-risk infants (Bradshaw et al., 2019).

Eye contact, joint attention, and touch synchrony are driven from birth by the motor-emotional system for the enjoyment of shared experience (Greenspan, 2002; Trevarthen and Delafield-Butt, 2013). Early motor and coordination deficits result in atypical control of eye movements, delays in development of gestures such as pointing, and associated impairment of joint attention (Landa et al., 2013). In neurotypical infants, touch synchrony, the coordination of affectionate touch with episodes of shared gaze, increases significantly from 3 to 9 months with the development of fine-motor skills. In this time, episodes of shared gaze decrease to about a third of the time, while shared attention to objects increases dramatically. This emphasizes the dynamic relationship between downstream development in the motor domain, which allows infants to crawl, grasp, and manipulate objects, and development of social competencies. ‘Sticky’ attention to objects is one of the earliest biomarkers of ASD, evident from 7 to 14 months. Difficulty disengaging attention interferes with social orienting and impairs social communication skills.

Speech comprehension relies on multi-sensory integration, predominantly auditory, enhanced by concomitant visual information; speech capacity relies on motor competence (McCleery et al., 2013; Akhtar et al., 2016; Stevenson et al., 2018).

The relationship between motor development in infancy and later cognition correlates neurally with the involvement of extensive cortico-subcortical networks and structures such as the dorsolateral prefrontal cortex and the neocerebellum in both motor and cognitive functions (Heineman et al., 2018).

Up to 87% of individuals with ASD demonstrate atypical sensory processing, often categorized as hyper-sensitive, hypo-sensitive, or sensory-seeking behaviors. From 6 months of age, signs of sensory processing abnormality are predictive of social communication deficits and repetitive behaviors in childhood, and ASD diagnosis (Robertson and Baron-Cohen, 2017).

Synchronous multisensory experiences, including proprioceptive, haptic, visual, and auditory, enhance neural connectivity and sensory-motor processing (Werchan et al., 2018). This paper hypothesizes that impoverishment of environmental stimulation, both social and non-social, chronic SNS-HPA hyperarousal, and disrupted parent-infant biobehavioral synchrony, result in trajectories of compensatory sensory hyper- or hypo-sensitivity and sensory seeking behaviors. In the same way that short-sightedness is a neuronal consequence of prolonged exposure to interior environments with suboptimal opportunities for long-distance focus, this paper proposes that the three variations of sensory processing difficulty are compensations within the global neuronal workspace for suboptimal motor and sensory-motor enrichment in very early life. Compensatory trajectories vary according to the environmental experience, predispositions, and feedback loops activated within that individual infant.

## Parent–Infant Biobehavioral Synchrony Optimizes Reciprocity Chains and Protects Stress Response Settings

### Biobehavioral Synchrony Integrates Psychosocial and Biological Models of Parent–Infant Interaction

Feldman’s theory of parent-infant biobehavioral synchrony integrates psychosocial and biological models of parent-infant co-regulation, in order to describe interactions which optimize secure psychological attachment and other long-term developmental outcomes (Feldman, 2007, 2016). This paper uses the term biobehavioral synchrony to refer to reciprocal motor and multi-sensory parent–infant exchanges, integrating behavioral observation and parent-reported psychosocial experience with neural and physiological correlates. Biobehavioral synchrony significantly expands upon the concept of synchrony used in social communication models of ASD etiology (Green et al., 2015). Biobehavioral synchrony stabilizes the myriad physiological, neural, and behavioral feedback loops that operate within the complex adaptive system of parent and infant.

To give one example, biobehavioral synchrony between a parent and newborn occurs during contact with a carer’s body and skin. This early proximity, known as skin-to-skin contact, comprises rich and complex sensory-motor stimulation for the infant, and supports both positive parent affect and early infant experience of secure psychological attachment. Skin-to-skin contact improves glucose and oxygen levels, downregulates the infant’s autonomic nervous system, including heart and respiratory rates, and improves breastfeeding outcomes due to sensory-motor activation of mammalian reflexes (Moore et al., 2016).

This paper offers an interdisciplinary synthesis of heterogenous studies investigating infant dysregulation, stress, and parent-infant biobehavioral synchrony. Infant dysregulation may be caused by, and may cause, disrupted parent–infant biobehavioral synchrony. Both parent and infant stress may disrupt parent–infant biobehavioral synchrony, and each may also result from disrupted parent–infant biobehavioral synchrony. In this way, disruptions to parent–infant biobehavioral synchrony in very early life may trigger a butterfly effect of multi-directional cascades of impaired cognitive, social, emotional and self-regulatory skills in ASD susceptible children.

#### Cued Care

In very early life, an infant signals or cues his or her biological needs, e.g., for milk, or for richer sensory-motor experience, through affect-driven motor behaviors and autonomic cues. Motor behaviors include spinal extension or writhing spinal movements, postural changes, grimaces, and non-speech-like vocalizations, including groans and cries. Autonomic cues include reddened face, increased respiratory rate, and tremors. An infant increases intensity of signaling along a spectrum of SNS-HPA arousal, from mild agitation to screaming.

‘Cued care’ has also been known as ‘responsive care’ or ‘attunement,’ and refers to parenting behaviors which respond to infant cues. Cued care is the foundational behavioral mechanism underlying parent-infant biobehavioral synchrony (Shonkoff and Phillips, 2000; Mansfield and Cordova, 2007; Fogel et al., 2008; Swain et al., 2013; Matas et al., 2016). The care-giver aims to downregulate the infant’s SNS-HPA axis and meet the infant’s needs for safety, nutrition, and loving human-mediated sensory-motor nourishment, by providing eye-contact, touch, motor interaction, and sounds, including speech and song, and also by providing non-human sensory-motor nourishment, such as opportunities for experience of the complex non-human natural environment. Milk and sensory-motor enrichment upregulate the parasympathetic nervous system (PNS), which downregulates the SNS. Downregulation of the SNS-HPA axis facilitates learning and joint attention, as well as cognitive and social processes (Greenspan, 2002).

Extinction of cues may occur when the infant does not receive adequate responses to his or her communications, a process elucidated in the operant conditioning principles of the first wave of the school of behaviorism (FWB), which has been widely applied in infant-care from the mid-twentieth century. The rate at which infant cues are extinguished may also depend on an infant’s underlying genetic or biological susceptibility.

Behaviorally hypo-aroused infants offer minimal cues and are at particular risk of cue extinction. That is, they may quickly respond to SNS-HPA hyperarousal with ‘learned helplessness.’ Minimal cues result in less parental engagement, shortened reciprocity chains, and less opportunities for joint attention, reinforcing the extinction of cues. For these behaviorally hypo-aroused infants, cued care from birth protects against cascades of disrupted development (Greenspan, 2002).

#### Reciprocity Chains

Reciprocity chains refer to back-and-forth exchanges which incorporate infant responses to parent-initiated communication as well as parental responses to infant-initiated cues. Behavioral reciprocity chains build on cued care, resulting in parent–infant biobehavioral synchrony. Cues from the infant, including affect-driven movements toward the parent, and sensory-motor and speech responses from the parent, including co-ordination of gaze, downregulate and organize the infant’s autonomic, motor, and attentive states. Reciprocity chains also facilitate lactation and downregulate parental stress responses, and are driven by the powerful evolutionary drive for mutual enjoyment.

Between the age of 2–3 months, specific combinations of interactive behaviors become more frequent and turn into constellations of behaviors. From a complexity perspective, these are ‘attractor states,’ recurring patterns that shape neural networks. In the second half of the first year of life, sensory-motor reciprocity chains are increasingly organized, symbolic, and complex, evolving into protophones and verbal communications, although the capacity to perceive and respond to complex non-verbal sensory-motor communication remains foundationally important for social communication throughout life.

Parent–infant biobehavioral synchrony, or enjoyment of increasingly long and complex sensory-motor reciprocity chains, is necessary from birth in order to protect cognitive, language, and social communication development (Britto et al., 2017). This paper proposes that very early life motor or sensory-motor lesions disrupt biobehavioral synchrony, either resulting in, caused by, or perpetuated by chronic SNS-HPA hyperarousal. The resultant shortened, less complex reciprocity chains trigger cascades of atypical sensory processing, motor skills, language, and cognition typical of ASD.

For example, at 4 months of age, the optimal interactive structure comprises a predominance of ‘infant-leads-parent-follows,’ but this is less likely to be found with high-risk siblings, even though maternal responsivity, scaffolding, and linguistic input are the same. By 6 months of age, high-risk siblings demonstrate attenuated social attention and fewer speech-like vocalizations. Although some parents may respond in the second half of the first year of life to minimal infant cuing with disengagement and decreased gaze, many parents of high-risk siblings become directive, in an effort to impose attentional shift and scaffold communicative behaviors. Unfortunately, either pattern may perpetuate a cascade of impaired social development in susceptible infants. ‘Undemanding synchrony,’ a pattern of noticing and responding to the infant’s cues without attempting to control, is the optimal parent response (Green et al., 2017).

### Chronic SNS-HPA Hyperarousal in Very Early Life Triggers Atypical Developmental Trajectories in ASD Susceptible Infants

The Avon Longitudinal Study of Parents and Children, a prospective longitudinal cohort study of the offspring of 14,541 pregnant women, demonstrates that high levels of irritable or unsettled infant behavior in very early life predicts autistic traits at 6 months and 2 years of age (Bolton et al., 2012). This association is corroborated by other longitudinal and retrospective studies (Box 2).

**BOX 2. Studies demonstrating links between unsettled behavior in very early life and ASD.**

In a 2013 audit of 208 preschool children with ASD, 50% of whom had intellectual disability, child health records demonstrated significantly more consultations for crying, feeding or sleeping problems regulatory problems in the first 18 months of life for children who were later diagnosed with ASD (Barnevik et al., 2013).

A 2017 retrospective study investigating 80 monozygotic twin pairs and 46 dizoygotic twin pairs with autistic traits and ASD found that early medical events, such as low birth weight or prenatal valproate exposure, and poor sleep, feeding problems, frequent vomiting, and crying in the first year of life, were associated with an increased risk of autistic traits and ASD. Cumulative load of risk factors correlated with severity of ASD. The authors concluded that non-shared environmental stressors, or allostatic load, resulted in epigenomic expression of autistic traits and ASD in individuals with genetic vulnerability (Willfors et al., 2017).

A 2018 analysis of the Danish National Birth Cohort showed that parents of the 973 children later diagnosed with ASD reported the same rates of periods of crying for over half an hour in the first 6 months of life as non-ASD children. However, when infants who had excessive crying in both groups were compared, those later diagnosed with ASD had cried longer per day and more days per week in the first 6 months than their excessively crying controls. Mothers who reported difficulty caring for their infant when interviewed at 6 months and 18 months post-birth were more likely to have a child later diagnosed with ASD (Lemcke et al., 2018).

In a 2018 retrospective comparative study of 200 children, parent-reported persistent infant crying, and excessive crying with long duration, that is, higher allostatic load, was associated with later diagnosis of ASD (Bag et al., 2018).

In a 2019 longitudinal sample of 282 high-risk siblings compared with 114 low risk infants, assessed at 6, 12, and 24 months of age, 6-month-old high-risk siblings later diagnosed with ASD exhibited poorer parent-reported regulatory capacity and less surgency compared to high-risk siblings and low risk infants not later diagnosed with ASD. Approach behaviors such as smiling and vocal reactivity were less. At 12 months, these temperament differences persisted, with high-risk siblings who were later diagnosed with ASD demonstrating decreased surgency or approach, impaired regulatory capacity, and increased negativity compared with typically developing infants (Paterson et al., 2019).

Motor development, as this paper argues, is primary for healthy development of sensory, social, and cognitive skills, and is facilitated by rich sensory-motor experience, including irregular postural variation. However, the benefits of rich sensory-motor experience occur when the newborn is in a quiet, alert state and able to integrate sensory feedback from primary and secondary generalized movements and other environmental experience (Hadders-Algra, 2018b). This paper hypothesizes that chronic SNS-HPA hyperarousal in very early life, most commonly associated with unsettled infant behavior, but sometimes with hypoaroused behaviors, as discussed below, disrupts capacity to maintain necessary attention on sensory feedback to achieve developmentally appropriate motor and sensory-motor learning tasks, including auditory and visual tasks, joint attention, and motor planning and sequencing (Bilgin et al., 2018; Breeman et al., 2018). These disruptions further perpetuate chronic SNS-HPA axis hyperarousal and add to allostatic load as the child continues to struggle with ongoing developmental tasks, until ASD is diagnosed.

The hypothesis that chronic SNS-HPA hyperarousal disrupts the capacity to attend and develop motor skills is corroborated by the Danish National Cohort study. Although this cohort showed no link between transient ‘infantile colic’ and developmental coordination disorder in later childhood, infants with persistent crying were at increased risk of developmental coordination disorder (Milidou et al., 2015). A prospective study of 37 infants with colic found transient developmental lags in both fine and gross motor skills at 6 months. They were less responsive and less able to maintain optimal functioning for months after resolution of the crying, though these subtle impacts resolved by 12–18 months of age (Sloman et al., 1990).

Corroborating the hypothesis that chronic SNS-HPA hyperarousal plays a key role in perpetuating disrupted feedback loops in ASD etiology, 4-month-old infants later diagnosed with ASD show altered respiratory sinus arrhythmia response to a social stressor compared to non-autistic infants. Similarly, toddlers later diagnosed with ASD have less variable respiratory sinus arrhythmia at 18 months of age (Sheinkopf et al., 2019). ASD adolescents report high levels of anger-focused rumination (Patel et al., 2018).

#### Hypoaroused Behavior

Although many children diagnosed with ASD are retrospectively reported to have had regulatory problems in the domains of feeds, sleep and cry-fuss problems in very early life, other children diagnosed with ASD are retrospectively characterized as ‘easy babies,’ whose behavioral regulation difficulties emerged in toddlerhood or later.

The Affect Diathesis model proposes that these behaviorally hypoaroused ASD-susceptible infants also experience chronic SNS-HPA hyperarousal, which is subjected to PNS override (Greenspan, 2002). In behaviorally hypoaroused babies, affect-driven sensory-motor cues are partially extinguished, either due to biological propensity or early ‘learned helplessness’ or both, resulting in less parental engagement and shortened reciprocity chains. These infants may be particularly vulnerable to the developmental impacts of suboptimal environmental stimulation and disrupted parent–infant biobehavioral synchrony (Middlemiss et al., 2012).

#### Atypical Crying Acoustics

One month old infants later diagnosed with ASD are reported by parents to have louder, more distressed, less typical cries, as if in pain (English et al., 2018). At 6 months of age, parents report that high-risk siblings produce cries with higher pitch and greater variability than low risk infants, as if in pain. High-risk siblings later diagnosed with ASD produced the widest frequency range and most poorly phonated cries. Parents report difficulty understanding the causes of crying episodes in the early life of infants later diagnosed with ASD (Esposito et al., 2017). Paradoxically, by 12 months of age, high-risk infants and infants later diagnosed with ASD have shorter cry durations, with higher frequency and decreased number of pauses compared to low-risk or neurotypical toddlers (Unwin et al., 2017). This paper proposes that environmental factors, that is, one or more problems of feeds, sleep and unmet needs for richer sensory-motor and social stimulation, may induce cries at the extreme end of the spectrum of distress in very early life, triggering aversive or avoidant responses in the care-giver, and finally, extinction of cries, associated with cascades of atypical development (Bag et al., 2018). In very early life, infant crying is commonly inappropriately attributed to gut pain (Bergmann et al., 2014; Gieruszczak-Bialek et al., 2015; Gordon et al., 2018).

#### Suboptimal Sensory-Motor Stimulation

The neurobiological model of infant cry-fuss problems argues that infants in a low sensory environment cry because of unmet needs for sensory-motor enrichment, in a biological bid to optimize development of neural circuitries during a window of critically sensitive neuroplasticity and monoaminergic excitability (Douglas and Hill, 2013a). The human infant’s biological requirement for rich postural variability and rich and complex sensory-motor reciprocity chains for normal motor development has been discussed previously. Interestingly, a study of infant macaques found that rich social environments in the first week of the macaques’ lives improved gaze-following and social skills at 7 months of age (Simpson et al., 2016).

Regulatory difficulties in very early life are typically interpreted, through a linear causative theoretical frame, as an early sign of sensory processing deficit in infants who later develop ASD. By 12 months, when neural changes are entrenched, sensory processing and regulatory reactivity are known to predict parent-reported executive function deficits in children later diagnosed with ASD (Robertson and Baron-Cohen, 2017; Stephens et al., 2018). But this paper applies the neurobiological model of cry-fuss problems to argue that infant dysregulation in very early life is less likely to be a sign of pre-existing sensory processing problems, and more likely to be a behavioral biomarker of chronic SNS-HPA hyperarousal resulting from environmental factors, including suboptimal sensory-motor stimulation, which predispose to sensory processing problems.

#### Feeding Dysregulation

Children diagnosed with ASD are 5 times more likely to have a feeding problem than neurotypical children (Peverill et al., 2019). In very early life, ‘feeding problems’ refer to fussing at the breast or bottle, and breast or bottle refusal. Infant feeding problems often result in a cascade of worsening parental anxiety, controlling parental feeding practices, and conditioned infant withdrawal or distress (Estrem et al., 2016). The latter is referred to in the neurobiological model of cry-fuss problems as ‘conditioned SNS-HPA hyperarousal’ with feeds, and may result in temporary re-setting of the HPA axis in the critically sensitive neural networks of very early life (Douglas and Hill, 2013a). Feeding-related signs such as refluxing or back-arching and fussing with the breast or bottle are often inappropriately medicalized, and underlying causes, such as positional instability with breastfeeds or conditioned SNS-HPA hyperarousal with breast or bottle feeds, remain untreated (Douglas, 2013). Feeding problems may escalate into a developmental trajectory of dysregulated feeding behavior into childhood (Winsper and Wolke, 2014). In the older child, the term ‘feeding problems’ encompasses a range of concerns, including food selectivity, problematic mealtime behavior, and oromotor challenges, and are most commonly the downstream effect of early life feeding problems. Children with feeding problems may withdraw from or reject their mother’s touch; their mothers touch them less; and shared eye contact is reduced (Estrem et al., 2016).

Feeding problems in children diagnosed with ASD decrease from time of diagnosis to clinically insignificance by school age, the same pattern followed by feeding problems in neurotypical children. However, ASD children with higher levels of anxiety or mood difficulties and externalizing behavioral challenges are most at risk of a chronic course of feeding problems, with bi-directional negative effects on family interactions, emphasizing the role of chronic SNS-HPA hyperarousal in perpetuation of atypical developmental trajectories (Peverill et al., 2019).

#### Sleep Dysregulation

There are strong links between sleep disruptions in the first months of life and impaired cognitive, sleep and behavioral outcomes at school-age (Williams et al., 2016, 2017). However, a prospective birth cohort of 5151 children in the Netherlands found that reports of sleep problems co-occur with autistic traits in early childhood beginning from 18 months of age, when parental reports of sleep problems are resolving for neurotypical infants (Verhoeff et al., 2018).

Children with ASD show more frequent ‘bedtime resistance,’ sleep anxiety, and longer durations of night waking than children with other development disabilities (Cohen et al., 2014; Valicenti-McDermott et al., 2019). A study of 1201 children with ASD demonstrates links between aggressive behavior and sleep disruption, and proposes that this link is mediated by chronic SNS-HPA hyperarousal, noting that children with ASD experience a great deal of stress resulting from impairments in their ability to communicate with others or to understand the world around them (Shui et al., 2018). Sleep difficulties worsen behavior and cognition problems for the ASD child, and impact negatively on his or her family’s communications.

This paper proposes that sleep problems in children with ASD are a consequence, first, of elevated levels of anxiety or chronic SNS-HPA hyperarousal, and, second, disrupted circadian rhythms, both worsened by the widespread application of ‘sleep training’ or FWB sleep strategies for children with ASD, which paradoxically disrupt the circadian clock.

Adults with ASD have dampened cortisol secretion and subjective somatic physiological arousal, associated with increased sleep onset latency, poorer sleep efficiency, and increased waking after sleep onset. This corroborates the hypothesis that dysregulation of the SNS-HPA is a key mechanism underlying phenotypic expression of ASD (Baker et al., 2019).

### Chronic SNS-HPA Hyperarousal Affects the Gut Microbiome and Metabolic and Immune Settings in ASD

The gut is a major microbial–host interface, and a dominant immune organ. Immune function, HPA axis regulation, and the microbiota-gut-brain axis interact and co-evolve in the mother-infant complex adaptive system, and are each affected by stress. The infant gut microbiome in very early life has life-long effect on the settings of metabolism, immune, endocrine, and gut health. Gut dysbiosis is associated with impaired gut barrier integrity, inflammation and autoimmune disease (Cenit et al., 2017; Warner, 2018).

Mothers of children with ASD are twice as likely to report at least one gastrointestinal symptom in their child between 6 and 36 months. High-risk siblings have a greater prevalence of gastrointestinal symptoms. Young children diagnosed with ASD demonstrate significant gut dysbiosis (Coretti et al., 2018). Chronic constipation is the most common gastrointestinal problem in children with ASD. Many children with ASD have functional gastrointestinal tract disorders relating to selective eating, medications, and differences in sensory processing, which are associated with increased anxiety (Tye et al., 2019). Gastrointestinal problems may worsen developmental cascades of child behavior problems and disrupted family interactions. Studies show a strong correlation between gastrointestinal dysfunction and autism severity, across all domains including speech, social and behavioral (Ding et al., 2017). Early clinical trials suggest that by targeting the gut ecosystem, both ASD and gastrointestinal tract symptoms can be impacted, suggesting potential shared mechanisms (Tye et al., 2019).

However, this paper proposes that widespread inappropriate medicalization of infant behavioral cues, usually as signs of gut problems, results in a failure to identify and manage the underlying environmental factors which precipitate chronic SNS-HPA hyperarousal in very early life. Inappropriate medicalization of upregulated behavior and the failure to identify and manage underlying clinical problems have deleterious effects on gut health long-term. Instead of applying a linear and causative disease model which assumes that gut dysbiosis causes excessive infant crying, the neurobiological model of cry-fuss problems proposes that chronic SNS-HPA hyperarousal and feeding problems, and also the widely prescribed proton pump inhibitors, interact in the complex adaptive system of the mother and infant, out of which gut dysbiosis emerges (Douglas P.S. and Hill P.S., 2011; Douglas and Hill, 2013a; Castellani et al., 2017; Rhoads et al., 2018). This paper proposes that these and other environmental factors disrupt parent-infant biobehavioral synchrony, precipitating multiple trajectories of atypical development in ASD susceptible infants.

Chronic inflammation is emerging as the critical pathophysiological feature of mental disorders generally, associated with dysregulation of normal microglial synaptic pruning (Cenit et al., 2017). Siniscalco hypothesizes that pro-inflammatory processes and immune alterations in very early life are etiological events for autism (Siniscalco, 2015) (Box 3). ASD has been linked with:

• Increased pro-inflammatory cytokines in the cerebro-spinal fluid
• Acquired mitochondrial dysfunction, an early sign of neurodegeneration
• Decreased antioxidants in urine
• Higher plasma GABA levels
• Amino acid and neuropeptide disruptions
• Increased autoimmune antibodies targeting central nervous system proteins.

**BOX 3. Evidence demonstrating that ASD is a pro-inflammatory state.**

Many autism susceptibility genes are localized in the immune system and related to immune or infection pathways (Carter, 2019), and ASD is associated with an increased prevalence of specific immune-related conditions (Tye et al., 2019). Endoplasmic reticulum stress, oxidative stress, and apoptosis have been proposed as molecular mechanisms underlying autism (Dong et al., 2018). The oxidative and integrated stress responses are upregulated in the autism brain and may contribute to myelination problems (Carter, 2019). Increased autoantibodies directed toward central nervous system proteins have been observed in children with ASD, which may signal heightened inflammatory processes or an autoimmune component that could decrease the integrity of the mucosal barrier (Tye et al., 2019). Cytokines are proteins produced by neurons that regulate immune responses including hematopoiesis, inflammation, immune cell proliferation and differentiation.

Pro-inflammatory cytokines are found in higher levels in children with more severe ASD symptoms compared to ASD children with milder symptom presentation (Tye et al., 2019). A recent meta-analysis of cytokine levels in unmedicated individuals with ASD, mostly children, confirmed an abnormal cytokine profile, characterized by elevations in proinflammatory cytokines and reduced levels of anti-inflammatory cytokines (Masi et al., 2014).

ASD is associated with altered expression of genes associated with blood-brain barrier integrity, increased neuroinflammation, and possibly impaired gut barrier integrity. Immunological abnormalities affect both the gastrointestinal system and microglial cells of the brain and CNS. Monocytes, the precursors for macrophages, dendritic and microglial cells, show significant pro-inflammatory dysfunctions in ASD children. Individuals with ASD have upregulated inflammatory cytokines which induce blood brain barrier disruption. Altered blood brain barrier permeability directly influences neural plasticity, connectivity and function, and is hypothesized to underlie impairments in social interaction, communication behavior (Siniscalco, 2015).

Chronic SNS-HPA hyperarousal releases cortisol, which alters intestinal motility, gut epithelial permeability, and induces changes in gut microbial composition. This paper argues that the pro-inflammatory state of children with ASD arises from chronic SNS-HPA hyperarousal, or stress and anxiety, with long-term multi-directional impacts upon gut health, behavior and immune trajectories (De Palma et al., 2014; Gottfried et al., 2015; Siniscalco, 2015; Ding et al., 2017; Carter, 2019).

Excessive infant crying, or chronic SNS-HPA hyperarousal, is a pro-inflammatory state, impacting on gut microbiome and permeability, with long-term effects on metabolic, immune and mental health in susceptible infants (Partty et al., 2017; Rhoads et al., 2018). The Affect Diathesis model theorizes that behaviorally hypo-aroused infants also experience chronic SNS-HPA hyperarousal, with PNS override, which would be expected to similarly predispose to pro-inflammatory states (Greenspan, 2002). Anxiety disorder is common in adults with ASD, and symptoms of anxiety cause substantial functional impairment in ASD adults more broadly (Tye et al., 2019). This paper proposes that chronic anxiety and gastrointestinal problems in children and adults diagnosed with ASD are a downstream effect of chronic SNS-HPA axis dysregulation that begins in very early life.

### Dysregulated Parent Mood Increases the Risk of ASD in Susceptible Children

A meta-analysis of nine observational studies shows increased ASD risk in children exposed to parental affective, depressive, and bipolar disorders (Ayano et al., 2019). Previous findings of links between maternal antenatal antidepressant use and ASD are attributed to the confounder of maternal mood disorder (Sujan et al., 2019). But there is, in particular, an increased risk of ASD in children of mothers who experience affective and depressive disorders, and this can be attributed to the developmental impacts of disrupted parent–infant biobehavioral synchrony in susceptible infants.

Depressed mothers with a 6-month-old baby show lower baseline vagal tone, linked with poorer emotional regulation and sensitivity to stress; less vagal brake, linked with poorer emotional regulation; less joint attention; and less initiation of interaction including touch and vocalization with the infant. Depressed mothers are less likely to touch the child, take significantly longer to respond to changes in their infant’s behavior, and have less capacity to repair interactive errors. A cycle of mutual disengagement occurs, as the infant develops flat affect and makes fewer bids for joint gaze and response. Matched states are more likely to be negative, in distress or anger. Difficulties in mother-infant biobehavioral synchrony are detected not only in cases of full-blown clinical depression but among mothers with chronic sub-clinical symptomatology (Tietz et al., 2014). An anxious mother may direct very active communications toward her baby, which are not attuned to the infant’s cues. The resultant shortened, less complex reciprocity chains between both highly anxious and depressed mothers and their infants in very early life predict the child’s emotional dysregulation in later life (Feldman, 2007).

## Biology-Culture Mismatch in Very Early Life Generates Environmental Factors Which Increase the Risk of ASD in Susceptible Infants

A susceptible infant experiences biology-culture mismatch as adversity. Although the human infant is highly adaptive across a wide variety of culturally determined infantcare practices, a significant gap between cultural practices and evolutionary expectation may result in chronic SNS-HPA hyperarousal in very early life, in addition to other risks (Barr, 1990, 1999; Hofer, 2002; Douglas, 2005; McKenna et al., 2007; Ball, 2008; Gettler and McKenna, 2011; Koss and Gunnar, 2018; Renz-Poster and De Bock, 2018). This paper proposes that the initial motor and sensory-motor neural lesions of ASD are more likely in susceptible children when environmental factors are mismatched with biological expectations during the critically neuroplastic, injury-sensitive first 100 days post-birth, resulting in chronic SNS-HPA hyperarousal which either triggers or perpetuates multi-directional cascades of atypical development.

In very early life, the infant evolved, in the *Homo sapien’s* environment of evolutionary adaptedness, to expect:

(1) Rich environmental stimulation, including
  (a) Prolonged physical contact with older children and adults, including co-sleeping and diverse and frequent social sensory-motor enrichment
  (b) High levels of postural variability
  (c) Multi-centric social interactions
  (d) Complex non-social environmental stimulation, e.g., outdoors.
(2) Affect-driven, increasingly long, sensory-motor reciprocity chains with caring older children and adults. Mutual positive affect between adults and infants, or enjoyment and delight, has ensured *Homo sapiens’* evolutionary survival.
(3) Human milk transferred directly from the lactating breast to the infant gut, optimizing the gut microbiome, and metabolic, endocrine, and immune protection. Frequent and flexible breastfeeding facilitates increasingly long and complex motor and sensory-motor reciprocity chains.

Currently, in very early life, parents receive a great deal of conflicting advice from health professionals concerning breastfeeding, infant sensory needs, unsettled infant behavior and parent-infant sleep, and consult with multiple providers (McCallum et al., 2011; Schmied et al., 2011). There are serious gaps in health professional training across disciplines in management of breastfeeding and infant behavior problems (Renfrew et al., 2012; Rimer and Hiscock, 2014; Kirby et al., 2015; Gavine et al., 2017); widespread inappropriate medicalization of infant behavior, risking worsened outcomes; and substantial evidence that popularly applied approaches to breastfeeding and infant regulatory problems do not help parents and babies, or make breastfeeding, crying and sleep problems worse (Douglas, 2013; Svensson et al., 2013; Bergmann et al., 2014; Gieruszczak-Bialek et al., 2015; Kempler et al., 2016; Thompson et al., 2016; Woods et al., 2016; NHMRC, 2017; O’Shea et al., 2017; Gordon et al., 2018).

That is, the gap between biological expectation and socioculturally determined advice concerning infantcare is profound in contemporary societies, placing susceptible infants at neurodevelopmental risk, and increasing the risk of maternal postnatal anxiety and depression (Stein et al., 2014; Dias and Figueiredo, 2015; Tsivos et al., 2015). Infant cry-fuss, feeding, and sleep problems are often highly stressful for parents, and predispose to maternal postnatal depression (Dorheim et al., 2009; Vik et al., 2009; Dias and Figueiredo, 2015).

This paper hypothesizes that three key environmental factors emerge from biology-culture mismatch, and interact in the complex adaptive system of the parent and infant, increasing the risk of ASD in susceptible infants: suboptimal environmental stimulation, disruption of parent-infant biobehavioral synchrony, and gut dysbiosis or feeding problems.

### Suboptimal Environmental Stimulation

In Australia and in many countries today, parents continue to be advised that sleep training or FWB approaches are necessary for optimal developmental outcomes and good sleep habits (Table 3). Infant sleep training emerged in the 1950s and 1960s when the first wave of the school of behaviorism (FWB) in psychology was applied to infantcare. Yet high level evidence demonstrates no decreased night waking or reliably improved maternal mood scores as a result of FWB interventions, and no improvement in developmental outcomes (Price et al., 2012; Bryanton et al., 2013; Douglas and Hill, 2013b; Mindell and Lee, 2015; Price et al., 2015; Kempler et al., 2016; NHMRC, 2017; Pennestri et al., 2018).

This paper proposes that application of FWB approaches to infant sleep in very early life impacts negatively on neurodevelopmental outcomes in ASD-susceptible infants, because FWB approaches decrease environmental stimulation in four ways:

(1) Parents are advised to teach the infant ‘good sleep habits’ by having the infant sleep in a cot or on an immobile surface, often in a quiet dim room with deliberately minimized visual stimulation, iteratively throughout the day. This regular recourse to a low sensory interior environment impoverishes sensory-motor experience, both social and non-social, and fails to offer susceptible infants adequate task demands for optimal development of skills in interpretation of sensory information, adaptation of movements in response to external stimuli, and organization of postural control.
(2) Stimulation is problematized. Parents are advised to avoid social and non-social ‘overstimulation.’
(3) Inadequate sensory-motor stimulation results in SNS-HPA arousal (crying and fussing), which parents are advised to interpret as ‘tired signs’ or ‘overstimulation,’ triggering more attempts to put the baby to sleep.
(4) Patterns of inadequate environmental enrichment may result in chronic infant SNS-HPA hyperarousal (Douglas and Hill, 2013a; Whittingham and Douglas, 2014; Ball et al., 2018).

### Disruption of Parent–Infant Biobehavioral Synchrony

Our environment of evolutionary adaptedness offered infants rich opportunities for increasingly long and complex motor and sensory-motor reciprocity chains across the two dominant sites of parent–infant transaction in very early life, feeds and sleep. Patterns of cued care are associated in cross-cultural studies with downregulation of both infant and parents’ SNS-HPA axes (Hamilton, 1981; Barr et al., 1991; St James-Roberts et al., 2006; Wolke et al., 2017). Parent–infant biobehavioral synchrony is disrupted by problems of crying and fussing, breastfeeding, and sleep.

#### Inappropriate Medicalisation of Infant Cry-Fuss Behaviors

The neurobiological model of cry-fuss problems proposes that chronic SNS-HPA hyperarousal, manifesting as crying and fussing in otherwise well infants, emerges out of a mismatch between environment of evolutionary adaptedness and various socioculturally determined environmental factors in contemporary life.

But overdiagnosis and overtreatment, including in children, is a growing global concern, and occurs commonly in very early life, with deleterious effects (Coon et al., 2014; Brownlee et al., 2017). Parents are often taught that their infant’s cry results from physical pain, e.g., from reflux, aerophagia-induced reflux, food allergies or intolerances, lactose intolerance, or from oral connective tissue restrictions (Bergmann et al., 2014; Gieruszczak-Bialek et al., 2015; O’Shea et al., 2017; Gordon et al., 2018; Kapoor et al., 2018). The resultant inappropriate medicalization risks unintended outcomes and perpetuates disruption to parent-infant biobehavioral synchrony (Table 2).

**TABLE 2:** Cry-fuss problems: popular sociocultural and clinical advice which disrupts parent–infant biobehavioral synchrony compared with Neuroprotective Developmental Care strategies which promote parent–infant biobehavioral synchrony.

**Infant cues**	**Most likely cause**	**Popular diagnoses or advice**	**Neuroprotective Developmental Care strategy**
Crying	Poor satiety	Normalize infant distress	Identify and manage underlying breastfeeding problems
Crying	Suboptimal sensory-motor stimulation	Teach to self-settle in cot in order to develop autonomous sleep; apply graduated extinction	Educate parents about infant’s biological need for rich sensory-motor nourishment including physical contact
Grizzling and crying	Suboptimal sensory-motor stimulation	Overtired or overstimulated;	Use two tools, satiety with milk or satiety of sensory nourishment, to downregulate infant
		place in low sensory environment;	
		avoid eye-contact and interaction;	
		teach to self-settle in cot	
Back-arching	Sign of protest	Oral ties, oesophagitis, reflux, wind pain or gas, colic, allergy	Use two tools of satiety with milk and satiety of sensory nourishment to downregulate infant; educate parents re appropriate spinal support for infants
Writhing, grunting, grizzling when lying in cot	Suboptimal sensory-motor stimulation	Oral ties, oesophagitis, reflux, wind pain or gas, colic, allergy	Offer sensory nourishment; educate re dialing up of SNS also activates gut

#### First Wave Behavioral Interventions for Parent–Infant Sleep

In response to emerging neuroscience and psychological attachment research, FWB approaches have adopted a discourse which emphasizes the importance of cued care for secure attachment and optimal mental health outcomes. Yet paradoxically, FWB sleep advice continues to actively disrupt cued care and biobehavioral synchrony, by advising parents to behave in directive and non-contingent ways, iteratively, day and night (Table 3). More extreme FWB strategies, such as minimizing eye contact and interaction at sleep-time throughout the days and nights, iteratively present a ‘still face’ to the infant, which is known to result in greater efforts by the infant to engage at first, before he or she withdraws from cueing (Bertin, 2006). Standard FWB approaches advise parents to not respond to SNS-HPA arousal, or to delay responses, or to respond but not as they believe the baby intends. Parents are advised to iteratively override the powerful biological cues of sleepiness after feeds by applying behaviors such as burping, holding upright, or wrapping, at the same time as they are instructed to achieve prescribed nap frequencies and durations.

**TABLE 3:** Sleep problems: popular sociocultural and clinical advice which disrupts parent–infant biobehavioral synchrony.

**First wave behavioral belief**	**First wave behavioral strategy**	**Disruption to parent–baby biobehavioral synchrony**
Teaching self-settling improves infant sleep		
	Don’t let baby fall asleep with breastfeed or bottle-feed	Overrides powerful biological cue of sleepiness
	Put baby down in cot drowsy but awake, even if baby grizzles and cries for a time	Ignores infant cue; interprets infant cue as ‘resisting sleep’; baby may be crying due to suboptimal sensory-motor nourishment
	Create sleep associations with cot, white noise, swaddling, music, low sensory environment	Sleep is under stimulus-control of sleep pressure, not ‘associations’; baby develops negative associations with sleep place and rituals, interpreted as ‘resisting sleep’
	Sleep in quiet dark room during day	Worsened night-waking after 2–3 weeks, due to disruption of circadian clock
Feed-play-sleep cycles make life more manageable for parents		
	Don’t let baby fall asleep with breastfeed or bottle-feed	Overrides powerful biological cue of sleepiness
	Put baby down in cot drowsy but awake	Baby cries due to suboptimal sensory-motor nourishment, interpreted as ‘resisting sleep’
	Space out feeds	Baby cries due to hunger; Undermines breastfeeding success
Baby needs a lot of sleep for optimal brain development		
	Sleep breeds sleep	Worsened night-waking after 2–3 weeks, due to disruption of circadian clock
	Achieve ‘second sleep cycle’ during day-time naps	Worsened night-waking after 2–3 weeks, due to disruption of circadian clock
	Sleep routines with estimates of time awake and ideal duration of sleep	Baby is expected to spend longer asleep than actually needs, disrupting the biological sleep regulators
Mustn’t let baby get over-tired		
	Prescribed list of ‘tired cues’	Disempowers parents by undermining confidence in their capacity to experiment and learn what they baby is cueing
	Put baby down at first ‘tired cue’	Promotes disruption of the biological sleep regulators, due to disruption of the circadian clock and inadequate sleep pressure
	Put baby to bed early at night (6–7 pm)	Promotes disruption of the biological sleep regulators, due to disruption of the circadian clock and inadequate sleep pressure
Mustn’t let baby get overstimulated		
	Avoid leaving house or engaging in play or social activity in lead up to sleep times	Baby may be cuing for richer sensory-motor nourishment, not tiredness
Baby who grizzles and cries is ‘resisting’ sleep		Baby may be cuing for richer sensory-motor nourishment, not tiredness

FWB approaches may exacerbate parental anxiety, which predisposes to poor sleep efficiency and postnatal depression (Blunden et al., 2016; Etherton et al., 2016). The parent trying to enforce sleep because she believes it necessary for her baby’s healthy development is likely to feel anxious and distressed when the baby ‘resists sleep’ multiple times a day; the infant ‘resisting sleep’ (that is, whose sleep pressure is not yet high enough for easy sleep) is repeatedly subject to the biological stress of a low sensory environment, resulting in chronic SNS-HPA hyperarousal and increased allostatic load. This cycle may place behaviorally hypo-aroused infants at particular risk.

The link between sleep problems in very early life and behavioral and sleep problems in later childhood may be, paradoxically, mediated by the widespread sociocultural and clinical application of sleep training or FWB strategies, resulting in cascades of both parent and infant chronic SNS-HPA hyperarousal (Simard et al., 2017; Williams et al., 2017; Ball et al., 2018). FWB approaches also disrupt the dyadic synchrony of the circadian clock, by promoting long blocks of sleep during the day (Thomas et al., 2014).

#### Unidentified and Unmanaged Breastfeeding and Unsettled Infant Behavior Problems Increase Risk of Maternal Postnatal Depression

Cry-fuss problems, poor maternal sleep efficiency, and breastfeeding problems are key modifiable risk factors for post-natal depression (Vik et al., 2009; Stein et al., 2014; Dias and Figueiredo, 2015; Tsivos et al., 2015; Werner et al., 2015). Each of these may arise from, or be exacerbated by, a mismatch between popular sociocultural and clinical approaches, and infant biology. Health professionals report inadequate training in management of breastfeeding and unsettled infant behavior problems, and often recommend approaches which have been shown not to help, or may even worsen these problems, with associated deleterious effects on parent–infant biobehavioral synchrony (Price et al., 2012; Bryanton et al., 2013; Douglas and Hill, 2013b; Rimer and Hiscock, 2014; Mindell and Lee, 2015; Price et al., 2015; Blunden et al., 2016; Etherton et al., 2016; Kempler et al., 2016; Thompson et al., 2016; Gavine et al., 2017; NHMRC, 2017; Pennestri et al., 2018).

There is widespread recognition of the importance of prevention of, or early detection and treatment of, perinatal anxiety and depression, the most common mental health condition post-birth. Winnicott proposed that a state of maternal preoccupation or heightened sensitivity develops toward the end of pregnancy and lasts throughout the postpartum period. From an evolutionary perspective, this heightened state supports a woman’s ability to anticipate her infant’s needs and to respond to her infant’s unique cues, and may include anxious, hypervigilant and intrusive thoughts about the infant. In contemporary contexts, often characterized by minimal social support, the same heightened state increases her risk of postnatal anxiety and depression if infant behavior problems emerge.

### Gut Dysbiosis and Feeding Problems

In the *Homo sapiens* environment of evolutionary adaptedness, the complex behavior of breastfeeding is the dominant site of parent-infant motor and sensory-motor interaction in very early life. The infant gut is part of the enteromammary immune system, an extension of the maternal gastrointestinal and immune systems facilitated by breastfeeding. Breastfeeding offers powerful protection of the gut microbiome, with positive impact upon immune and metabolic health.

Successful breastfeeding is linked with increased amounts of cued care (Brown and Lee, 2013), which leads to longer and more complex reciprocity chains. Early breastfeeding is driven by infant stepping, suck and swallow movements (secondary variable movements) and complex sensory-motor interactions, offering rich and diverse environmental stimulation and opportunities for affect-driven sensory-motor reciprocity chains. This may explain associations between breastfeeding and higher IQ (Belfort, 2017). Cued care also improves breastfeeding duration (Brown and Arnott, 2014). Breastfeeding difficulties, and lactation-related breast pain predispose women to postnatal depression, with an associated cascade of related crying behaviors and disruption of mother–infant biobehavioral synchrony (Howard et al., 2006; Brown et al., 2015; Dias and Figueiredo, 2015).

Breastfeeding, either exclusively or with some formula supplementation, is associated with decreased risk of ASD (Schultz et al., 2006; Boucher et al., 2017; Tseng et al., 2017; Bittker and Bell, 2018; Manohar et al., 2018). A Spanish multicenter birth-cohort study of 1,346 children showed that longer duration of breastfeeding protected against autistic traits (Boucher et al., 2017). The Danish National Birth cohort study of 76,322 mothers showed that breastfeeding for less than 6 months was associated with an increased risk of diagnosis of ASD (Lemcke et al., 2018). High-risk siblings are more likely to be weaned earlier. Weaning after 6 months and exclusive breast milk are also associated with protection against gastrointestinal tract symptoms, in particular constipation and abdominal distress, in high-risk siblings (Penn et al., 2016).

This paper proposes that unidentified clinical breastfeeding problems such as positional instability or conditioned dialing up at the breast, may cause chronic SNS-HPA hyperarousal, premature weaning, and related gut dysbiosis (Table 4). Unfortunately, popular clinical approaches often don’t help, or may even worsen breastfeeding problems (Svensson et al., 2013; Thompson et al., 2016; Woods et al., 2016; O’Shea et al., 2017). Developmental trajectories of feeding problems and disrupted parent–infant biobehavioral synchrony may result (Douglas and Hill, 2013a).

**TABLE 4:** Breastfeeding problems: popular sociocultural and clinical advice which disrupts parent-infant biobehavioral synchrony compared with Neuroprotective Developmental Care strategies which promote parent–infant biobehavioral synchrony.

**Breastfeeding: infant cue**	**Most likely cause**	**Popular diagnoses or advice**	**Neuroprotective Developmental Care strategy**
Difficulty coming onto the breast	Positional instability, breast tissue drag, landing pad encroachment	Oral ties, oesophagitis, reflux, wind pain or gas, colic, allergy	Optimize fit and hold to optimize positional stability (gestalt breastfeeding)
Back-arching and pulling off at the breast	Positional instability, breast tissue drag, landing pad encroachment	Oral ties, oesophagitis, reflux, wind pain or gas, colic, allergy	Optimize fit and hold to optimize positional stability (gestalt breastfeeding)
Dialing up at the breast	Positional instability, breast tissue drag, landing pad encroachment	Oral ties, oesophagitis, reflux, wind pain or gas, colic, allergy	Optimize fit and hold to optimize positional stability (gestalt breastfeeding)
Dialing up whenever approaches breast or during breastfeeding (‘oral aversion’)	Conditioned hyperarousal (dialing up) of SNS, often secondary to positional instability but persisting once fit and hold are corrected	Oral ties, oesophagitis, reflux, wind pain or gas, colic, allergy	Comprehensive intervention for conditioned hyperarousal of SNS
Marathon feeds or excessively frequent feeds	Poor milk transfer	Oral ties, oesophagitis, reflux, wind pain or gas, colic, allergy	Optimize fit and hold to optimize milk transfer (gestalt breastfeeding)
Falls asleep at the end of a breastfeed	Normal biological process (↑ parasympathetic nervous system response, ↑ oxytocin, ↑ cholecystokin)	Allows bad habits or sleep associations to develop	Parents educated about healthy function of the biological sleep regulators

## Targeting Key Environmental Factors for Pre-Emptive Intervention

A prospective cohort study of 85,176 children in Norway shows that folic acid supplementation, taken by the mother from 4 weeks prior to 8 weeks after the first day of her last menstrual period, is associated with a lower risk of ASD. For this reason, folate supplementation from pre-conception is recommended as an initial step in pre-emptive intervention (Suren et al., 2013).

Pre-emptive intervention for ASD then aims to promote infant neuro-resilience by preventing, or re-stabilizing, disrupted feedback loops emerging from small motor and sensory-motor structural or functional lesions in very early life. Drawing on the preceding review of heterogeneous interdisciplinary research literature, the following will require optimization:

(1) Environmental enrichment, social and non-social (that is, ‘rich motor and sensory-motor nourishment’)
(2) Parent–infant biobehavioral synchrony, which is promoted by
  (a) Cued care for downregulation of the SNS-HPA axis
  (b) Multi-lateral strategies for management of cry-fuss problems
  (c) Avoidance of inappropriate medicalization of infant behavior
  (d) Healthy regulation of parent-infant sleep
  (e) Increasingly long and complex, multi-sensory, affect-driven reciprocity chains, often infant-initiated
  (f) Optimal parent mental health, which requires
  (i) Effective intervention for the key modifiable risk factors of breastfeeding, sleep and cry-fuss problems
  (ii) Strategies for managing difficult thoughts and feelings
(3) Gut microbiota and mitigation of pro-inflammatory states, promoted by
  (a) Optimizing breastfeeding
  (b) Teaching paced bottle-feeding
  (c) Avoiding inappropriate medical diagnoses.

Over the past 20 years, robust peer-reviewed theoretical frames have been developed for a program known as Neuroprotective Developmental Care (NDC or ‘the Possums programs’), which targets these environmental factors as anticipatory guidance in the antenatal period and as clinical and educational support throughout the first 6–12 months of life. NDC has applied an integrative, primary care generalist’s lens to heterogenous evidence from the fields of neuroscience, evolutionary biology, developmental psychology, and the medical, lactation and sleep sciences (Douglas P. and Hill P., 2011; Douglas P.S. and Hill P.S., 2011; Whittingham and Douglas, 2014; Douglas and Keogh, 2017; Douglas and Geddes, 2018). NDC is a novel, community-based, parent-mediated, early life clinical and educational intervention. The term ‘Neuroprotective Family Centered Developmental Care’ has been previously applied to Heideleise Als pioneering work in the hospital setting with prematurely born infants, demonstrating improved development outcomes in this population (Als, 2009; Altimier and Phillips, 2016).

NDC has been delivered clinically in the Australian primary care setting since 2011 and is also available as online programs and for interdisciplinary health professional education.

### Environmental Enrichment

This paper accepts the primacy of motor lesions *in utero*, intra-partum, or in very early life in ASD etiology. Pre-emptive intervention therefore advocates adult-mediated motor and sensory-motor environmental enrichment post-birth, both socially and non-socially, if early motor deficits are to be prevented or early disruption of feedback loops stabilized. Patterns of rich postural variability, with general movements of the infant’s limbs and trunk occurring against the adult’s changing bodily configurations (rather than in the context of long periods on an immobile cot or mattress), and patterns of rich multi-centric social interactions, that is, physically interactive play and enjoyment with older children and adults, are required to optimize sensory-motor feedback (Lickliter, 2011).

The NDC sensory domain is unique amongst infant-care programs in de-problematizing sensory stimulation, and offers parents multiple practical strategies for environmental enrichment, also referred to as healthy sensory-motor nourishment. Physical contact with the infant is encouraged where this is sensibly possible, which helps address the infant’s biological need for complex and continuing postural variation. NDC supports the primary carer to leave the low sensory environment of the Western interior daily for social contact, activities, and tasks, including frequent walks, as carers focuses on creating a rich, full and meaningful life, in large part outside the home, and to have the infant sleep day or night in the vicinity of the carer.

### Optimizing Parent–Infant Biobehavioral Synchrony

The first step to optimal parent–infant biobehavioral synchrony is the elimination of clinical disruptors, which allows attention to the small communications of the infant to emerge. The second step is to encourage attention to the present moment and prioritize playful enjoyment of the baby.

#### Multi-Domain Management of Cry-Fuss Problems

NDC applies a systematic 5-domain approach to management of cry-fuss problems in parents, developed from the neurobiological model of cry-fuss problems (Douglas and Hill, 2013a). Each of the domains (feeds, sleep, environmental enrichment, infant gut and health considerations, and parental mood) integrate multiple evidence-based strategies. Preliminary evaluation of the 5-domain approach shows halved crying and fussing durations (Douglas et al., 2013). NDC applies a ‘dialing up’ and ‘dialing down’ metaphor, referring to up-regulation and down-regulation of the SNS-HPA, to describe parents’ co-regulatory efforts as they respond to their infant’s cues (Douglas and Hill, 2013a).

#### Healthy Regulation of the Infant’s Circadian Clock and Sleep–Wake Homeostat

The NDC sleep domain comprises the Possums Sleep Program, which enriches environmental stimulation, both social and non-social, by offering the only existing evidence-based alternative to FWB approaches for parent-infant sleep (Whittingham and Douglas, 2014; Ball et al., 2018; Douglas, 2018). Preliminary evaluation confirms the Possums Sleep Program has high levels of acceptability to parents and improves their quality of life (Ball et al., 2018). Parents are educated about maintenance of healthy function of their baby’s sleep regulators, and offered evidence-based information which minimizes anxiety about sleep. This includes:

(1) Educating parents about normal sleep variability and normal night-waking trajectories
(2) Differentiating between excessive night waking and normal night waking
(3) Identifying circadian clock disruption or satiety problems that result in unnecessary disruption of parent-baby sleep,
(4) Removing disruptors to sleep efficiency for parents and baby
(5) Education about how to optimize the function of the two biological sleep regulators, the circadian clock and the sleep–wake homeostat.

#### Strategies for Managing Difficult Thoughts and Feelings

NDC aims to change parents’ relationship with difficult thoughts and feelings in order to optimize enjoyment of the baby, which results in increasingly long and complex reciprocity chains of interaction. Traditional Cognitive Behavioral Therapy, which has been extensively investigated perinatally and is demonstrated as effective, promotes disputing irrational thoughts, which in the perinatal context may paradoxically worsen the biological predisposition to maternal rumination, precipitating worsened anxiety and depression (Monteiro et al., 2018). For this reason, NDC integrates applied functional contextualism, popularly known as Acceptance and Commitment Therapy (ACT), a third wave behaviorism. ACT has a burgeoning evidence-base for depression and anxiety, overtaking traditional Cognitive Behavioral Therapy on some indicators, with preliminary positive findings for parenting (Blackledge and Hayes, 2006; Ruiz, 2010; Whittingham et al., 2014).

ACT avoids unnecessary pathologizing of mental health challenges. It helps parents clarify values, and targets rumination, which predisposes mothers to perinatal anxiety and depression and which has been thought to mediate impaired infant development outcomes (DeJong et al., 2016). ACT offers a range of strategies for cognitive defusion and expansion of attention, in order to help parents manage difficult thoughts and feelings. ACT teaches behavioral activation skills, in the service of values, demonstrated to be effective with postnatal anxiety and depression (Lavender et al., 2016; Marchesi et al., 2016), and mindfulness, or anchoring in the present moment, demonstrated to assist in anxiety and depression generally (Gotink et al., 2015). ACT offers acceptance-focused processes and self-compassion, also demonstrated to help prevent postpartum depression and anxiety (Monteiro et al., 2018). A study of 139 parents of children with ASD showed that those who demonstrated more self-compassion experienced less stress and improved quality of life (Bohadana et al., 2019).

ACT is vitally concerned with context, and is well-suited for integration into health professionals’ consultations for common infantcare problems post-birth. ACT strategies are integrated into the Possums 5-domain approach to cry-fuss problems, the Possums Sleep Program, and gestalt breastfeeding (Whittingham and Douglas, 2016; Douglas and Keogh, 2017). Within these programs, women with moderate to severe mental illness are appropriately diagnosed, treated pharmaceutically as needed, and referred for ongoing psychological support.

#### ‘Growing Joy in Early Life’

Unlike early intervention programs derived from social communication models, NDC does not instruct parents to avoid intrusiveness or directiveness, or offer suggestions about the meaning of infant cues and how to respond, which may inadvertently communicate assumptions of parental incompetence and may also increase parental anxiety. Similarly, NDC also does not employ tools such as the Neonatal Observation Scale to teach parents about their infant’s behavioral repertoire and communication competence in the first six months of life (Nugent, 2013). Instead, NDC educates parents about the benefits of reciprocity chains, and encourages enjoyment of the baby. NDC proposes that parental competence and evolutionary drive for enjoyment of the baby will emerge in families once disruptive sociocultural and clinical advice are removed, underlying clinical problems are identified and repaired, and the importance of satisfying and socially engaged days outside the home explained.

NDC confidence in parental competence is corroborated by a study of 864 parent–newborn pairs observed spending time together as the baby lay close to the parent. Parents were given minimal instructions, but asked to interact with the baby comfortably, as they saw fit. Most of the 480 full-term newborns showed subtle affect-driven initiation of arm movements toward the parents as they interacted, though this was somewhat reduced in the prematurely born infants, and all parents engaged in quiet and supportive interaction without being intrusive (Delafield-Butt et al., 2018).

### Optimizing Breastfeeding and Enjoyment of Feeds

Optimizing gut health in susceptible infants requires not only avoidance of unnecessary medicalization of unsettled infant behavior, but importantly, the support of breastfeeding success, with its known gut and immune protections.

A systematic review of ultrasound studies of breastfeeding mother-baby pairs forms the biomechanical basis for a new ‘gestalt’ model of clinical breastfeeding support, out of which the NDC gestalt breastfeeding approach is developed (Douglas and Geddes, 2018). Gestalt breastfeeding builds on the pioneering advance of baby-led breastfeeding, which emphasizes cued care by activating and responding to the baby’s mammalian reflexes, but points to the evidence showing that this is not enough to achieve pain-free effective milk transfer for many women (Svensson et al., 2013; Woods et al., 2016). The gestalt approach educates about the biomechanics of effective breastfeeding. Impaired fit and hold and the resultant infant cues of fussing at the breast, difficulty coming onto the breast, back-arching, maternal nipple pain, marathon feeds, excessively frequent feeds, excessive night waking, and poor weight gain, are addressed applying gestalt methods (Douglas and Keogh, 2017; Douglas and Geddes, 2018).

Formula, which offers the human infant unphysiological doses of cow’s milk protein, lacks a myriad immune factors, micro-nutrients, and live bacteria, predisposing to short- and long-term gut dysbiosis and increased risk of immune system alterations. DNA methylation, a key mechanism of epigenomic regulation, is highly responsive to diet. Nutrients and bioactive compounds are known to alter the expression of genes at the transcriptional level and result in long-term phenotypic changes (Barker et al., 2018).

However, women feed babies in complex contemporary sociocultural contexts, facing multiple disruptors, pain, and conflicting clinical advice. NDC is strictly non-judgmental concerning mode of infant feeding, and teaches paced bottle-feeding in order to optimize feeding-related reciprocity chains in formula or expressed-breastmilk-fed infants. NDC also identifies and manages conditioned dialing up with the bottle, which is commonly inappropriately medicalized (Table 5).

**TABLE 5:** Bottle-feeding problems: popular sociocultural and clinical advice which disrupts parent–infant biobehavioral synchrony compared with Neuroprotective Developmental Care strategies which promote parent–infant biobehavioral synchrony.

**Bottle-feeding: infant cue**	**Most likely cause**	**Popular diagnoses or advice**	**Neuroprotective Developmental Care strategy**
Back-arching and fussing	Positional instability	Oral ties, oesophagitis, reflux, wind pain or gas, colic, allergy	Paced bottle-feeding
Back-arching and fussing	Does not want more milk; pressure on feeds due to spacing	Oral ties, oesophagitis, reflux, wind pain or gas, colic, allergy	Paced bottle-feeding
Back-arching and fussing	Conditioned hyperarousal of SNS	Oral ties, oesophagitis, reflux, wind pain or gas, colic, allergy	Paced bottle-feeding, health professional support to build enjoyable feeding associations

## Conclusion

ASDs are complex polygenic syndromes displaying highly variable phenotypic expressions and co-morbidities. Myriad environmental factors are known to modulate epigenomic regulation of ASD phenotypic expression. By 6 months of age, when the first signs of atypical connectivity emerge in neuroimaging studies, neuropathy may be entrenched. This paper responds to a recent call from the International Society for Autism Research for the development of theoretical foundations for, and clinical translation of, pre-emptive intervention for ASD.

The first 100 days of human life are characterized by injury-sensitive neuroplasticity, correlating with in the neural biomarker of cortical subplate persistence and the behavioral biomarker of the crying diathesis. Environmental factors in these critically injury-sensitive first 100 days may either initiate or perpetuate structural or functional lesions in global neuronal workspace of susceptible infants. This paper builds on ground-breaking etiological models concerning the primacy of motor lesions in ASD etiology, to propose that chronic SNS-HPA hyperarousal and disrupted parent-infant biobehavioral synchrony are key mechanisms facilitating perpetuation of the atypical developmental trajectories of ASD, triggered by very early motor and sensory-motor lesions.

Existing clinical guidelines for protection of infant growth and development in very early life, including support for feeds and sleep, are confused and conflicting, exacerbating parental anxiety. From an evolutionary perspective, popular clinical and cultural advice often promotes biology-culture mismatch, resulting in chronic SNS-HPA hyperarousal and disrupted parent–infant biobehavioral synchrony. Health professionals are not adequately trained in the identification and management of common clinical problems arising in the domains of feeds, sleep, cry-fuss problems and parental mood, which disrupt parent–infant biobehavioral synchrony. This health system context places ASD susceptible infants, and infants susceptible to other neurodevelopmental disorders, at risk.

Any pre-emptive intervention for ASD that aims to apply a focused selection of ‘active ingredients’ risks exacerbating parental anxiety, because parents already receive conflicting and confusing advice concerning common infant-care problems. Contact with these providers will continue. This paper argues that a comprehensive, holistic, community-based approach to the care of at-risk siblings is necessary to support parents who must inevitably navigate the health system, and proposes an Australian program known as NDC.

Research funding for evaluation of primary care initiatives remains scarce and is diminishing. Evaluation of NDC as pre-emptive intervention for ASD (Box 4), using real world methodologies suitable for complex community-based interventions, is required.

**BOX 4. When to apply NDC as pre-emptive intervention.**

Families with the following risk factors for ASD should be offered NDC pre-emptive intervention as soon as possible, beginning with anticipatory antenatal education and throughout the first 6 months of life:

• Child diagnosed with ASD
• Parent diagnosed with ASD.

Further potential indicators for NDC pre-emptive intervention are perinatal factors associated with later diagnosis of ASD, including:

• Antenatal depression
• Pre-eclampsia
• Premature birth
• Caesarian section under general anesthesia
• Infant hypoxic-ischemic encephalopathy (Chien and Lin, 2015; Getahun et al., 2017).

The following factors that have been associated with infant neurodevelopmental vulnerability also warrant NDC as pre-emptive intervention:

• Unsettled infant behavior
• Atypical crying acoustics
• Breastfeeding or feeding difficulties
• Maternal postnatal depression
• Plagiocephaly (marker of increased risk of transient motor and developmental delay, suggesting need for enriched sensory-motor experience)
• Other signs of developmental vulnerability (Martiniuk et al., 2017; Renz-Poster and De Bock, 2018)

If efficacy of NDC is demonstrated in infants at risk, a second phase would evaluate NDC as a health system response, replacing current conflicting and confusing advice from multiple health professionals in order to offer consistent, evidence-based care which optimizes neuroprotection for all parents and infants from the antenatal period onward, universally available through usual maternal and child health services.

## Author Contributions

The author confirms being the sole contributor of this work and has approved it for publication.

## Conflict of Interest

PD is the medical director of a small primary care charity, Possums Education, which sells Neuroprotective Developmental Care programs online for parents, and also as education workshops for health professionals www.possumsonline.com. All revenue raised is returned into the development of education and research.

## Abbreviations

ASD: Autism spectrum disorder/s
FWB: First wave behaviorism/first wave behavioral
PNS: Parasympathetic nervous system
NDC: Neuroprotective Developmental Care
SNS: Sympathetic nervous system
SNS-HPA: Sympathetic nervous system and hypothalamic-pituitary-adrenal axis
